# Role of the Lipid Environment in the Dimerization of Transmembrane Domains of Glycophorin A

**Published:** 2015

**Authors:** A. S. Kuznetsov, P. E. Volynsky, R. G. Efremov

**Affiliations:** M.M. Shemyakin and Yu. A. Ovchinnikov Institute of Bioorganic Chemistry, Russian Academy of Sciences, GSP-7, Miklukho-Maklaya Str., 16/10, 117997, Moscow, Russia; Higher School of Economics, Myasnitskaya Str., 20, 101000, Moscow, Russia; Joint Supercomputer Center of Russian Academy of Sciences, Leninskiy Pr., 32a, 119991, Moscow, Russia

**Keywords:** transmembrane domain, glycophorin A, molecular dynamics, protein-protein interactions, role of the lipid membrane, free energy of intermolecular interactions

## Abstract

An efficient computational approach is developed to quantify the free energy of a spontaneous association of the α-helices of proteins in the membrane environment. The approach is based on the numerical decomposition of the free energy profiles of the transmembrane (TM) helices into components corresponding to protein-protein, protein-lipid, and protein-water interactions. The method was tested for the TM segments of human glycophorin A (GpA) and two mutant forms, Gly83Ala and Thr87Val. It was shown that lipids make a significant negative contribution to the free energy of dimerization, while amino acid residues forming the interface of the helix-helix contact may be unfavorable in terms of free energy. The detailed balance between different energy contributions is highly dependent on the amino acid sequence of the TM protein segment. The results show the dominant role of the environment in the interaction of membrane proteins that is changing our notion of the driving force behind the spontaneous association of TM α-helices. Adequate estimation of the contribution of the water-lipid environment thus becomes an extremely urgent task for a rational design of new molecules targeting bitopic membrane proteins, including receptor tyrosine kinases.

## INTRODUCTION

Protein-protein interactions play an important role in the formation of the supramolecular complexes that perform the essential functions in the cell. The study of these interactions is particularly difficult in the case of membrane proteins (MPs), as they lose their native properties in a nonmembranous environment. Since the α-helix is the most common structural element in the transmembrane (TM) domains of MPs, in some cases, the study of protein-protein interactions is reduced to the consideration of the behavior of TM α-helices in the membrane. This behavior determines the spatial structure of the membrane-bound fragments of ion channels [1-4] and functioning of biotopic (i.e., having only one TM-helix) membrane proteins: in particular, receptor tyrosine kinases (RTKs) [5-9]. It was shown that activation of RTKs requires the formation of dimers or oligomers, wherein the association process involves TM domains interaction [6, 10-12]. Disruption of MPs leads to severe diseases, such as diabetes mellitus or cancer; so, their study is particularly important. It was shown that a number of disorders in RTK functions result from mutations in the TM domains [13-16]. Designing therapeutic agents selectively acting on the TM-segments of the target protein referred to as “interceptor” peptides is a challenging endeavor. However, resolving this problem requires a detailed understanding of all the molecular mechanisms of signal transmission through these domains of target RTKs [17-20]. Thus, the interaction of α-helices in the membrane is a key process which requires a detailed study.

Experimental methods for studying the interaction of TM domains may include the use of hybrid molecular biological structures with marker-proteins, for example, FRET [21] and TOXCAT [22], as well as the determination of the spatial structure of dimers by nuclear magnetic resonance spectroscopy in media that mimic the membrane environment [ 5, 23-25]. Both types of approaches yield good results; however, they are associated with complex and long-term expression and production of target proteins, as well as with difficulties in stabilizing oligomeric states in membrane-like environments. Therefore, computational simulation techniques which efficiently evaluate the parameters of protein complexes are increasingly used to deal with the problem. In particular, molecular dynamics (MD) is used to quantify the free energy association in studying the role of the medium and the effect of mutations on the dimerization of the TM domains of membrane proteins [11, 12, 26, 27].

Based on the results of an analysis of the TM domains amino acid sequences of several MPs, in particular glycophorin A (GpA), the concept of “dimerization motifs,” i.e., specific residues that are located in the contact area and determine the interaction between α-helices, has been proposed [28-31]. The so-called glycophorin dimerization motif includes two glycine residues separated in the sequence by the other three residues and is designated as GG4. This motif is also found in other proteins [5], but in some cases it can be nonfunctional [9]. Glycophorin A is still being considered a good model for the theoretical and experimental study of the influence of point mutations and the properties of the medium on the dimerization of TM helices [27, 29, 32-34]. Despite the fact that in these works a key role in dimer formation is assigned to protein-protein interactions, it is shown that the parameters of the lipid membrane influence dimerization [35-37]. These differences were attributed to the condition of hydrophobic mismatch, wherein the most efficient incorporation of a protein into the membrane is ensured by the fact that the length of the membrane-spanning segment of a protein must be equal to the hydrophobic thickness of the lipid bilayer [38].

It is known that the lipid membrane is not a homogeneous medium and tend to form clusters of lipids even in the simplest model systems [39, 40]. Proteins, in turn, often contain binding sites on their surface for the molecules of phospholipids and cholesterol which can modulate their activity [4, 21, 41, 42]. Incorporation of any protein into the membrane changes the properties of the latter one [43, 44], and the dimerization process may induce more complex effects [40, 45]. Thus, the interaction of the TM domains of MP is not limited only to the search for the most favorable protein-protein contacts, but also represents a complex combination of the contributions and interactions of the proteins and the medium.

Therefore, the question arises as to how to calculate the contribution of membrane environment effects to the free energy of TM domains dimerization of a protein. Moreover, it is necessary to identify the role of each amino acid residue in the process. Important information can be gleaned by studying the effects of point mutations in the amino acid sequences on the distribution of contributions to the energy of the protein and the environment. In the present study, we have investigated the effects of two mutations in the TM domain of GpA on the formation of a dimer. We studied substitutions that influence different factors of dimerization: the mutation Gly83Ala disrupting compact folding of helices and Thr87Val mutation that interferes with the formation of the intermolecular hydrogen bond. The molecular dynamics method was used for the study at the atomic level in an explicit zwitterionic lipid bilayer. The results allow us to draw a conclusion about the dominant role of the membrane in the initial stage of dimer formation and the different molecular mechanisms of disruption of TM complexes association in mutant proteins.

## MATERIALS AND METHODS

**The systems**

For the study, we chose two mutations affecting amino acid residues, which are the most important for glycophorin A TM domain dimerization: Gly83Ala and Thr87Val [46, 47]. The amino acid sequences of the peptides are shown in *Table 1*. Monomers and dimers of the TM domain were studied in the hydrated lipid bilayers of palmitoyloleylphosphatidylcholine (POPC). In the monomers, the protein was represented as an ideal α-helix; the initial conformation of the dimer was built on the basis of the experimental structure of the wildtype GpA dimer (PDB ID: 2KPF [48]). Models of dimers of mutant GpA forms were built similarly, substituting the corresponding residues, followed by energy relaxation of the structure. The models of the protein were placed into a lipid bilayer (128 molecules of POPC), and water molecules were added using *genbox *utility. The size of the calculated cell was 6.5 × 6.5 × 7.5 nm^3^.

**Table 1:** Amino acid sequences of the studied peptides

Peptide	Amino acid sequence
GpA	SEPEITLIIFGVMAGVIGTILLISYGIRR
GpA Thr87Val	SEPEITLIIFGVMAGVIGVILLISYGIRR
GpA Gly83Ala	SEPEITLIIFGVMAAVIGTILLISYGIRR

*Note.* Amino acid substitutions are underlined. Italics show terminal residues; their influence was not considered in calculation of the energy profiles for protein-protein interaction.

**Molecular dynamics**

MD trajectories were calculated using the GROMACS software package version 4.6.7 and GROMOS96 43a2 force field [49]. The integration time step was 2 fs. Periodic boundary conditions applied in all directions. The calculations were carried out at a constant pressure of 1 atm and a temperature of 315 K. The system pressure was controlled using a Berendsen barostat algorithm [50]. The V-rescale thermostat algorithm was employed for the protein, lipids, and water [51]. Electrostatic interactions were treated using Ewald summation and van der Waals interactions using the Lennard-Jones potential truncated at a cut-off distance of 1.0/1.2 nm.

Before calculating the MD trajectory, energy minimization of the system was performed using the steepest descent method (50,000 steps) and then during the first 50 ps the MD temperature in the system was increased linearly from 5 to 315 K. For relaxation of the membrane environment, a trajectory of 5 ns with a fixed protein molecule was first calculated, followed by 50 ns MD run without any restrictions. The stability of the dimer was analyzed from the change of the crossing angle formed between α-helical axes and the inclination relative to the plane of the bilayer, change of the secondary structure, as well as the value of the root-mean-square deviation (RMSD) of the coordinates of protein backbone atoms from their initial values.

**Calculations of the free energy of α-helices dimerization**

Profiles of the free energy of TM-domains association of the receptor were obtained by integrating the mean force acting between the monomers. The distance between the mass centers of the peptides was used as the reaction coordinate. A range of dimer states characterized by different distances between the monomers was generated for subsequent MD simulations (32 points, from 0.75 to 2.10 nm with 0.05 nm increments). These initial states were obtained by parallel transfer of monomers at a predetermined distance along the line passing through their mass centers. The membrane was relaxed *via *50-ns MD, and the length of the production run was also 50 ns. For each of the states, the value of the mean force acting between the protein monomers was calculated and integrated. In the resulting energy profiles, we evaluated the standard error by defining five independent non-overlapping fragments of each MD trajectory. Two independent calculations were performed for each system; the total length of the accumulated MD trajectories was about 10 μs.

**Decomposition of the interaction energy of α-helices**

The total energy profile was decomposed into components according to the following approach: at each MD step forces were recalculated using the coordinates of the atoms, taking into account only the sub-systems of interest. Further, these forces were averaged over the length of the MD trajectory, projected on the direction corresponding to the reaction coordinate, and integrated. For plotting the distribution of individual amino acid residues contributions, we selected energy values corresponding to the global minimum in the total profile of the potential of mean force.

## RESULTS AND DISCUSSION

**Stability of the peptides in the lipid bilayer**

As shown by MD, all models of the TM-domains of GpA are stable in terms of the investigated parameters. Thus, the α-helices in the central part were folded and the crossing angles between the axes of α-helices in dimers were very close to the initial values (*Table 2*). In the case of monomers, we observed a helix inclination relative to the membrane normal caused by the adjusting of the peptide to the environment, referred to as a hydrophobic mismatch. In all systems, there was a partial unfolding of the helices ends at the contact areas with water. The intermolecular hydrogen bond, which is regarded as one of the important factors that stabilize the dimer, was formed by Thr87 residues in the wild-type dimer GpA and its Gly83Ala mutant. However, the dimer with the mutation Thr87Val, which makes this bond impossible, was stable in MD and did not differ significantly by other parameters.

**Table 2:** Stability of GpA dimers in MD simulations. The root-mean-square deviation (RMSD) of the resulting structure from the initial one, crossing angle and secondary structure change

Structure	RMSD from the initial structure, nm*	Crossing angle of α-helices axes,degrees	Content of α-helix conformation,%
Initial	0.0	-40 ± 2	84 ± 2
GpA	2.9 ± 0.2 (1.5 ± 0.1)	-39 ± 3	74 ± 2
GpA Thr87Val	2.4 ± 0.3 (1.7 ± 0.3)	-57± 4	73 ± 2
GpA Gly83Ala	1.9 ± 0.4 (1.3 ± 0.5)	-47 ± 7	77 ± 2

*RMSD was calculated based on the backbone atomic coordinates; the value for Ile73-Ile95 residues is given in parenthesis.

**The free energy of GpA α-helices dimerization**

**Fig. 1:**
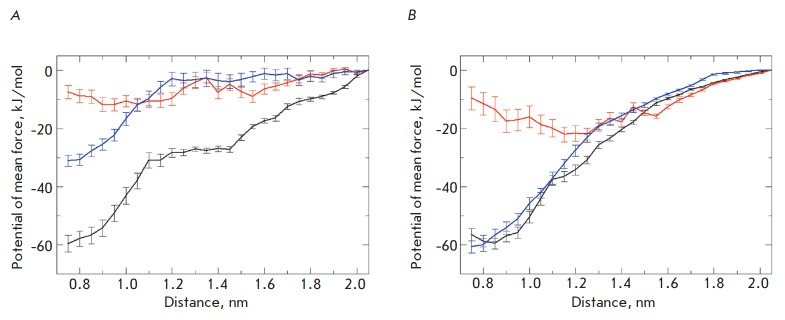
Profiles of the free energy of TM-domains dimerization of wild-type GpA (black) and its mutant forms, Thr87Val (red) and Gly83Ala (blue). (A): total free energy profiles; (B): contribution of protein-protein interactions for the central part of α-helices (residues Ile73-Ile95)

The obtained profiles of the association energies of all three systems under study indicate the presence of a stable dimeric state of each protein, but the minimum energy depths are different (*Fig. 1A*). Thus, mutant Thr87Val has a very small free energy of dimerization (in absolute value) (-16 ± 3 kJ/mol) compared to the wild-type dimer (-60 ± 3 kJ/mol). Mutation Gly83Ala also weakens the dimerization, but not too much (-30 ± 5 kJ/mol). Thus, the behavior of the studied peptides varies. For a more detailed study of the differences, we quantified similar energy profiles of a direct protein- protein interaction without the contributions of lipids and water (*Fig. 1B*). It was found that the mutant Gly83Ala drastically differs in energy from the wildtype dimer and reveals an energy profile of the same shape and depth, while the mutant Thr87Val is also considerably weaker in this case. Thus, the mutation Gly83Ala affects the interaction of the GpA TM-domain with the lipid environment rather than the direct contact of the monomers. When comparing the curves, it is noticeable that in the case of total energy profiles, minima are shifted toward shorter distances compared with the profiles characterizing protein-protein interactions. Thus, the membrane “brings” the monomers in closer contact compared to their equilibrium position, without including the medium effects.

**Contribution of amino acid residues**

**Fig. 2:**
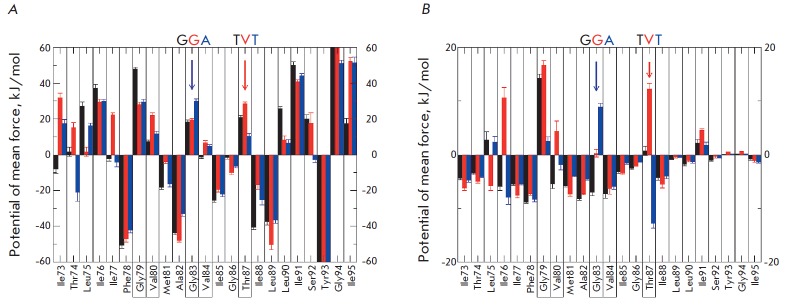
Diagrams of amino acid residues contributions to the free energy of dimerization for wild-type GpA (black) and its mutant forms, Thr87Val (red) and Gly83Ala (blue). (A): contributions to the total free energy; (B): contributions of protein-protein interactions. Color arrows point to the mutated residues. Frames show amino acid residues that form helix-helix contacts in the dimer

A detailed study of the distribution of energy contributions from the residues showed that the residues which lie at the dimerization interface can form energetically unfavorable contacts (Gly79, Val80, Gly83, Thr87), while the main contribution that promotes the formation of the complex is made by residues that interact with the membrane (Phe78, Ala82, Ile89, Tyr93) (*Fig. 2A*). The influence of the Thr87Val mutation is seen in protein-protein interactions, while the Gly83Ala mutation has in this case a compensation effect (*Fig. 2B*). Thus, the influences of the two mutations differ: substitution of Thr87Val disrupts protein-protein interactions, making it impossible for a hydrogen bond to be formed, while Gly83Ala leads to a minor rearrangement in the structure destabilizing the interaction of the dimer with the membrane environment. It should also be noted that the Gly83 residue in the dimer does not interact with lipids and indirectly affects the entire structure, improves overall packing in protein-protein contacts (*Table 2*, *Fig. 1B*), but decreases the interaction with the membrane environment. Thus, in describing the interaction of α-helices in the membrane, it is insufficient to consider just the protein-protein interactions and packing density in the protein structure. The lipid environment can make an equal contribution to the stabilization of the dimeric form: so, it is important to study the effects of TM-peptides of different nature on the membrane.

## CONCLUSIONS

Results of atomistic molecular dynamics simulations of the interaction between glycophorin A TM domains and two mutant forms show that the lipid membrane plays an important role in dimer formation, together with the direct contact between the monomers. One of the main conclusions in this work is that the interactions between residues lying on the TM helix-helix interface are often energetically unfavorable. This is, however, compensated by favorable contributions of the protein–environment contacts to the total free energy of the system. We propose two different scenarios of the disruption of GpA TM-helices association caused by point mutations. Thus, the mutation Thr87Val directly disrupts the protein-protein interactions, and the substitution Gly83Ala has an indirect effect, affecting the membrane environment of the receptor. Thus, the water-lipid environment actively participates in the functioning of the receptor systems of the cell, and its role should be taken into account when considering the functions of membrane proteins, as well as for a rational design of new molecules modulating the function of signaling systems, primarily receptor tyrosine kinases, and other membrane proteins in a goal-oriented manner.

## Abbreviations

GpA: human glycophorin A
MP: membrane proteins
MD: molecular dynamics
POPC: palmitoyloleylphosphatidylcholine
RMSD: root-mean-square deviation
RTK: receptor tyrosine kinase
TM: transmembrane
